# Long COVID: a cross-sectional study of respiratory muscle strength, lung function, and persistent symptoms at one year after hospital discharge

**DOI:** 10.36416/1806-3756/e20240246

**Published:** 2024-11-16

**Authors:** Ellys Rhaiara Nunes Rebouças, Taynara Rodrigues Ramos, Barbara Galdino de Sousa, Rayana Fialho da Costa, Samara Sousa Vasconcelos Gouveia, Italo Caldas Silva, Daniela Gardano Bucharles Mont’Alverne, Nataly Gurgel Campos

**Affiliations:** 1. Programa de Pós-Graduação em Fisioterapia e Funcionalidade, Universidade Federal do Ceará - UFC - Fortaleza (CE) Brasil.; 2. Grupo de Pesquisa Inspirafisio, Universidade Federal do Ceará - UFC - Fortaleza (CE) Brasil.; 3. Programa de Pós-Graduação em Ciências Médicas, Departamento de Medicina Clínica, Universidade Federal do Ceará - UFC - Fortaleza (CE) Brasil.; 4. Faculdade de Fisioterapia, Universidade Federal do Delta do Parnaíba -UFDPar - Parnaíba (PI) Brasil.; 5. Departamento de Fisioterapia, Universidade Federal do Ceará - UFC - Fortaleza (CE) Brasil.

## TO THE EDITOR:

Currently, the severity of and mortality associated with COVID-19 have been decreasing. However, there are a large number of survivors, who can have prolonged and varied symptoms, a condition known as post-acute COVID-19 syndrome, or “long COVID”, defined as the persistence of symptoms (not explained by another diagnosis) at 12 weeks after infection, whether those symptoms developed during or after the period of infection.^1^ ^-^ ^3^

The severity of the acute illness, measured by the number of symptoms, severity score, or hospital admission, can influence the risk of long COVID, greater severity translating to a higher risk. Fatigue, dyspnea, chest pain, and impaired quality of life are among the most commonly reported late symptoms of SARS-CoV-2 infection. The predilection of the virus for lung tissue is evident, with considerable consequences for the respiratory system in cases that require hospitalization.^4^ Approximately 94% of hospitalized patients have persistent lung parenchymal findings on chest CT scans at hospital discharge, and data from previous coronavirus infections (with the original severe acute respiratory syndrome coronavirus or the Middle East respiratory syndrome coronavirus) have suggested that there is substantial fibrotic post-COVID-19 sequelae, with systemic and biopsychosocial repercussions.^5^

The objective of this study was to assess respiratory muscle strength, lung function, and persistent symptoms in individuals hospitalized with COVID-19, at one year after discharge.

This was a cross-sectional study with a quantitative approach to data, conducted in the semi-intensive care unit of a reference hospital for infectious diseases and in the Cardiorespiratory Physiotherapy Laboratory of the Federal University of Ceará, both in the city of Fortaleza, Brazil. Participants were recruited between August and September of 2020. Persistent symptoms, respiratory muscle strength, and lung function were assessed at one year after hospital discharge. This study was approved by the Human Research Ethics Committee of the Federal University of Ceará (Reference no. 38197020.1.0000.5044), in accordance with Brazilian National Health Council Resolution no. 466/12.

We included individuals who were at least 18 years of age and had a clinical condition consistent with COVID-19 and a positive laboratory test result for SARS-CoV-2 infection, according to the diagnostic criteria established by the Brazilian National Ministry of Health.^6^ Those who were transferred to other (internal or external) hospital units during their hospital stay were excluded, as were those who were not discharged from the follow-up unit and those with previous neurological impairment.

In the first stage of data collection, we collected demographic and clinical characteristics of the patients during hospitalization, such as age, gender, BMI, previous comorbidities, and lifestyle habits (smoking and drinking), as well as the diagnosis, history, and hospitalization of the individual. In the second stage, at one year after hospital discharge, the patients were invited to complete a self-report questionnaire designed to collect information on the appearance of symptoms after COVID-19. Participants also underwent an assessment of respiratory muscle strength and lung function with manometry and spirometry, respectively.

The study sample included 50 participants, the majority (64%) of whom were male. The mean age was 54.86 ± 17.87 years, and the mean BMI was 28.85 ± 4.85 kg/m^2^. Of the 50 participants, 29 (58%) had preexisting comorbidities, cardiovascular and metabolic diseases being the most common, collectively accounting for 18%, and 23 (46%) were alcohol drinkers. The average length of hospital stay was 11 ± 8.8 days. Admission to the ICU was required in 10 cases (20%), and the mean ICU stay was 6.22 ± 8.04 days. Alterations were seen on CT scans in all 50 cases. Twenty-nine individuals (58%) required oxygen at admission and 8 (16%) were submitted to invasive mechanical ventilation. These data are shown in Table 1.

**Table 1:** Demographic, anthropometric, and clinical characteristics of the study participants.

Variable	(N = 50)
Age (years), mean ± SD	54.86 ± 17.87
Weight (kg), mean ± SD	78.7 ± 15.47
Height (m), mean ± SD	1.65 ± 0.09
BMI (kg/m^2^), mean ± SD	28.85 ± 4.85
Gender, n (%)	
Male	32 (64)
Comorbidities, n (%)	
Cardiovascular and metabolic	09 (18)
Cardiovascular	07 (14)
Metabolic	04 (08)
Respiratory	04 (08)
Infectious	02 (04)
Neurological	02 (04)
Alcohol-related	23 (46)
Hospital stay (days), mean ± SD	11 ± 8.8
ICU admission required, n (%)	10 (20)
ICU stay (days), mean ± SD	6.22 ± 8.04
Oxygen required at admission, n (%)	29 (58)
High-flow therapy, n (%)	04 (08)
Mechanical ventilation required, n (%)	08 (16)
Days on mechanical ventilation, mean ± SD	13.25 ± 6.06
Use of an NBA for more than 24 h, n (%)	06 (12)
Change on CT, n (%)	
< 50%	41 (82)
≥ 50%	9 (18)
NBA: neuromuscular blocking agent.	

The most recurrent persistent symptoms were respiratory, reported by 78% of the participants, with fatigue on slight exertion being the most prevalent (in 32%). At one year after hospital discharge (Table 2), the mean FVC was 3.27 ± 0.88, which was only 59.78% of predicted.

**Table 2:** Persistent symptoms, muscle strength, and lung function at 1 year after COVID-19.

Variable	(N = 50)
Cardiac symptoms, n (%)	
Palpitation and hypertension	8 (16.0)
Palpitation	5 (10.0)
Hypertension	5 (10.0)
Respiratory symptoms, n (%)	
Fatigue on slight exertion	16 (32.0)
Fatigue on medium exertion	9 (18.0)
Fatigue on heavy exertion	8 (16.0)
Persistent cough	6 (12.0)
Musculoskeletal symptoms, n (%)	
Muscle fatigue/weakness	12 (24.0)
Muscle/joint pain	10 (20.0)
Muscle pain, fatigue, and weakness	5 (10.0)
Dermatological symptoms, n (%)	
Dermatitis	11 (22.0)
Hair loss	10 (20.0)
Hair loss and dermatitis	2 (4.0)
Neurological symptoms, n (%)	
Memory loss	14 (28.0)
Memory loss and concentration deficit	9 (18.0)
Limb paresthesia	5 (10.0)
Loss of smell and/or taste	2 (4.0)
Psychological/emotional symptoms, n (%)	
Anxiety	9 (18.0)
Anxiety and depression	8 (16.0)
Irritability/stress	7 (14.0)
Pulmonary variables	
Manometry, mean ± SD	
MIP (cm/H_2_O)	112.80 ± 36.91
MIP (% of predicted)	103.46 ± 33.19
MEP (cm/H_2_O)	113.00 ± 32.67
MEP (% of predicted)	95.94 ± 25.4
Spirometry, mean ± SD	
FVC (L)	3.27 ± 0.88
FVC (% of predicted)	59.78 ± 16.89
FEV_1_ (L)	2.6 ± 0.88
FEV_1_ (% of predicted)	85.84 ± 31.84

MIP: maximal inspiratory pressure; and MEP: maximal expiratory pressure.

The findings related to the assessment of respiratory muscle strength and lung function revealed that all of the study participants had MIP and MEP values within the normal ranges. However, in the population studied, FVC was below the predicted values. At one year after the acute infection, the persistent symptoms most commonly reported were respiratory symptoms, with fatigue on slight exertion being the most commonly reported; musculoskeletal symptoms, such as fatigue and muscle weakness; and noticeable neurological symptoms, mainly memory loss.

Patients who have been infected with Sars-CoV-2, whether or not they required hospitalization, can develop long COVID, characterized by persistent COVID-19 symptoms that last more than four weeks after infection and are not explained by any alternative diagnosis. Of those who require hospitalization, at least 34% of survivors report persistent severe symptoms and organ dysfunction after discharge. These symptoms might, in part, be a consequence of the cytokine storm experienced in the acute phase of the infection.^7^ ^,^ ^8^ These long-lasting syndromes occur among patients with severe symptoms, but have also been reported regardless of the severity of the acute phase, hospitalization, and oxygen support.^9^

Recent studies have reported a prevalence of pulmonary function abnormalities after the acute phase of COVID-19. Although SARS-CoV-2 infection has repercussions on all systems, the lungs are the main organs affected, and damage can occur due to inflammatory mechanisms and direct action of the virus, leading to diffuse alveolar damage and pulmonary vascular thrombosis.^10^ In our study sample, the mean FVC was lower than predicted. These results are consistent with those of a study evaluating 186 COVID-19 survivors, in which FVC was significantly lower among the patients with persistent dyspnea, and a restrictive ventilatory pattern was observed in 47%.^9^

This study was carried out at a regional reference hospital for the treatment of COVID-19 and provides relevant findings on the manifestations of COVID-19, specifically persistent symptoms related to respiratory muscle strength and lung function, even after a long period of acute infection, enabling a comprehensive reflection on the impacts of the disease, through patient accounts. A limitation of this study is the small sample size, given the difficult-to-control conditions of the second wave of the COVID-19 pandemic.

In conclusion, patients hospitalized with COVID-19 seem to present FVC results below the predicted values one year after discharge. Among such patients, the most common persistent symptom appears to be fatigue on slight exertion.
